# Supplementary material for: Systematic review and meta-analysis on the effect of continuous subjective tinnitus on attention and habituation
Source: PeerJ. 2021 Nov 26;9:e12340. doi: 10.7717/peerj.12340 (PMC8628620; doi:10.7717/peerj.12340)
Supplement: Supplemental Information 3 [file peerj-09-12340-s003.docx]

| **S. No** | **Author and Year** | **Reviewer 1**  **(GS)** | **Reviewer 2**  **(BR)** | **Reviewer 3**  **(HP)** | **Decision** | **Reason for rejection**  **(reviewer 4 - HV)** |
| --- | --- | --- | --- | --- | --- | --- |
| 1 | Andersson 2000 | High | Low | Low | ACCEPT | NIL |
| 2 | Andersson 2005 | Very high | High | High | REJECT | High ROB |
| 3 | Araneda 2015 | Very low | Very low | Very low | ACCEPT | NIL |
| 4 | Asadapour 2016 | Very high | Moderate | Very high | REJECT | High ROB |
| 5 | Attias 1993 | Moderate | Low | Moderate | ACCEPT | NIL |
| 6 | Attias 1996 | Moderate | Low | Low | ACCEPT | NIL |
| 7 | Campbell 2018 | High | Low risk | Very high | REJECT | High ROB |
| 8 | Campbell 2019 | High | Low risk | Moderate | REJECT | High ROB (4^th^ RV – High) |
| 9 | Cuny 2004 | High | Moderate risk | Moderate | ACCEPT | NIL |
| 10 | DaS 2012 | High | High | High | REJECT | High ROB |
| 11 | Dornhohoffer 2006 | Very high | Low | High | REJECT | High ROB |
| 12 | Santos Filha | Very high | Low | High | REJECT | High ROB |
| 13 | Gabr | Very high | Very low | High | REJECT | High ROB |
| 14 | Haab 2014 | Very high | Moderate | High | REJECT | High ROB |
| 15 | Heeren | Moderate | Very low | Very low | ACCEPT | NIL |
| 16 | Holdefer 2013 | V,high | Moderate | High | REJECT | High ROB |
| 17 | Hong 2016 | Low | Low | Low | ACCEPT | NIL |
| 18 | Houdayer 2015 | Low | Low | Low | ACCEPT | NIL |
| 19 | Jackson 2014 | Very high | Very low | Moderate | ACCEPT | 4^th^ reviewer - Moderate |
| 20 | Jacobson 1996 | High | Moderate | Very high | REJECT | High ROB |
| 21 | Jacobson 2003 | High | Low | High | REJECT | High ROB |
| 22 | Kropp 2012 | Moderate | Low | High | REJECT | High ROB (4^th^ RV- High) |
| 23 | Li | High | Moderate | Very high | REJECT | High ROB |
| 24 | Lima 2019 | Moderate | Low | High | REJECT | High ROB (4^th^ RV- High) |
| 25 | Mahmoudian 2013 | Low | Low | Low | ACCEPT | NIL |
| 26 | Mannarelli 2017 | Low | Low | Low | ACCEPT | NIL |
| 27 | Mohebbi 2018 | Low | Low | Low | ACCEPT | NIL |
| 28 | Rossiter | Very high | Moderate | Low | ACCEPT | 4^th^ reviewer – Moderate |
| 29 | Shirashi T | Moderate | Moderate | Moderate | ACCEPT | NIL |
| 30 | Stevens 2007 | High | Moderate | Low | ACCEPT | 4^th^ reviewer – Moderate |
| 31 | Trevis 2016 | High | Low | Low | ACCEPT | NIL |
| 32 | Waechter 2015 | Moderate | Moderate | Moderate | ACCEPT | NIL |
| 33 | Zuraida 2016 | Very high | Low risk | Very high | REJECT | High ROB |

Note: ROB- Risk of Bias and RV- Reviewer
