# Supplementary material for: Systematic review and meta-analysis on the effect of continuous subjective tinnitus on attention and habituation
Source: PeerJ. 2021 Nov 26;9:e12340. doi: 10.7717/peerj.12340 (PMC8628620; doi:10.7717/peerj.12340)
Supplement: Supplemental Information 5 [file peerj-09-12340-s005.docx]

**Systematic review and meta-analysis on the effect of continuous subjective tinnitus on attention and habituation**

**1. Rationale for conducting the systematic review**

Attention and habituation play a vital role in the persistence and maintenance of tinnitus. To date, reviews addressing attention & habituation in tinnitus have been addressed as part of cognition and not as a separate entity. Further, the inclusion of studies with multifaceted tinnitus characteristics makes the findings difficult to generalize to a heterogenous population like tinnitus. In addition, it is well known that the hearing loss can mask the actual effect of tinnitus on attention. Existing reviews on attention and tinnitus includes both the studies that have and have not controlled for hearing loss. With respect to habituation, there were no reviews on the effect of tinnitus on habituation. Hence, a systematic review that focusses on the effect of continuous tinnitus on attention and habituation was conducted.

**2. The contribution that the systematic review makes to knowledge in light of previously published related reports.**

Pooled findings from behavioral and electrophysiological (MMN & P300) measures indicate that, there is an attentional impairment in individuals with continuous tinnitus, although, it is inconclusive whether few sub-domains or the entire domain of attention is affected. Further, a meta-analysis conducted on studies which have controlled for hearing shows a lack of clear attention impairment in tinnitus individuals. These results suggest the attentional effect can vary drastically based on whether the hearing is controlled or not, and we have higher number of studies in literature which have occasionally ignored hearing. In terms of habituation, there are very few studies with low risk of bias to draw any clear conclusions. This review necessitates the need to assess habituation in tinnitus population either by creating new paradigms or modifying the existing paradigms.
